# Drug-Induced Urinary Stone of Atazanavir Incidentally Found in an Asymptomatic Patient: A Case Report

**DOI:** 10.1155/2023/4890711

**Published:** 2023-03-29

**Authors:** Maëlle Plawecki, Marie Bistoquet, Pierre-Edouard Grillet, Nicolas Abdo, Jean-Sébastien Souweine, Jean-Paul Cristol

**Affiliations:** ^1^Department of Biochemistry, CHU Montpellier, Montpellier, France; ^2^PhyMedExp, University of Montpellier, INSERM U1046, CNRS UMR 9214, Montpellier University Hospital, Montpellier, France; ^3^Department of Infectious Diseases, CHU Montpellier, Montpellier, France; ^4^Department of Urology, CHU Montpellier, Montpellier, France

## Abstract

A HIV-infected female treated with a combination of emtricitabine/elvitegravir/tenofovir since 2017 presented an acute renal failure during her hospitalization for a SARS-CoV2 pneumonia. A computed tomography demonstrated left ureterohydronephrosis and ureteral stone. Fragments extracted by ureteroscopy showed a calculus composed of atazanavir and calcium oxalate. The patient's medical history showed atazanavir intake during ten years and then discontinued in 2017. This case report emphasizes that drug-induced urolithiasis should be considered when renal function declines, even far from discontinuation of atazanavir and without clinical signs of renal colitis. Moreover, identification of risk factors should alert to the possibility of drug-induced nephrolithiasis.

## 1. Introduction

Drug-induced urolithiasis represents 1% of all kidney stones. Protease inhibitors (PIs) are widely used in combination with other antiretroviral molecules for the treatment of human immunodeficiency virus (HIV). PI drugs are responsible for uncommon side effects. One concern associated with PIs, and especially atazanavir, is the appearance of crystalline precipitation in urine or renal tissue, leading to urolithiasis and renal dysfunction [1]. Only a few proportion of patients treated with atazanavir develop calculi (1%), suggesting that other factors contribute to the occurrence of this adverse effect [2]. In this paper, we report the presence of a urinary stone of atazanavir incidentally found in a patient showing no clinical evidence of renal colitis.

## 2. Case Presentation

**Figure 1:**
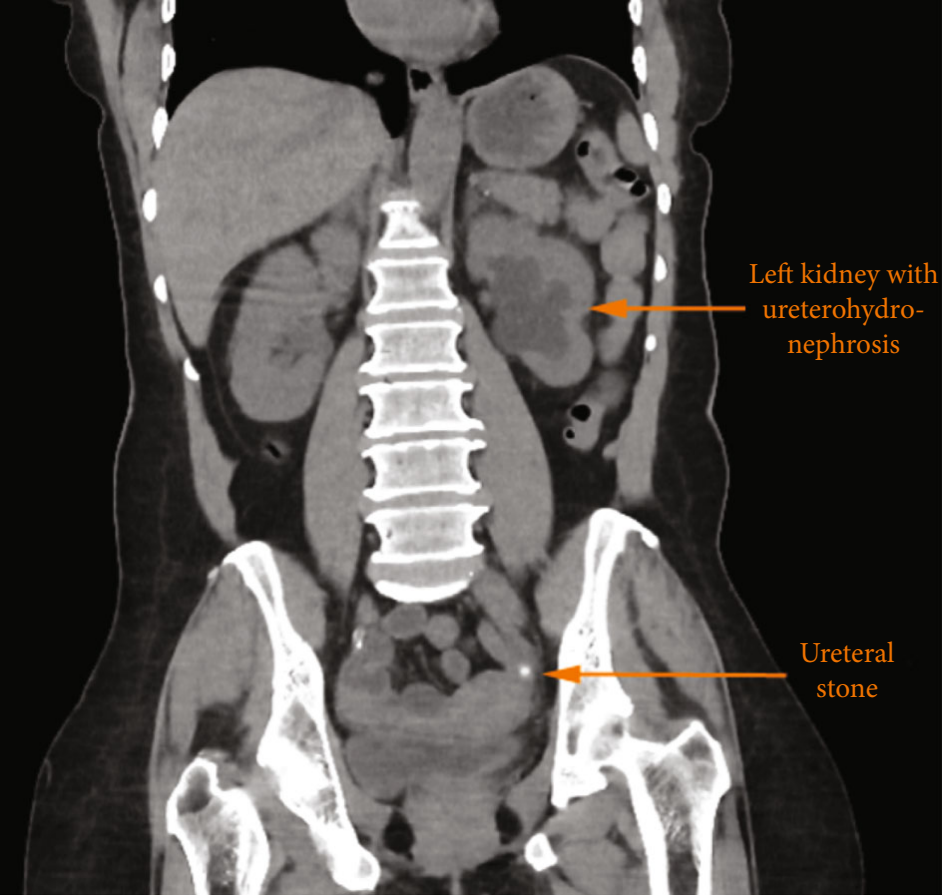
Abdominal computed tomography showing a hypotrophic left kidney with ureterohydronephrosis and a ureteral stone.

A HIV-infected female patient with a medical history of several episodes of functional acute renal failure was referred to the hospital for a SARS-CoV2 pneumonia which required oxygen therapy. She presented comorbidities as type 2 diabetes, dyslipidemia, arterial hypertension, and chronic kidney disease. Additionally, a combination of emtricitabine/elvitegravir/tenofovir was given since 2017 as antiretroviral treatment with a good tolerance and undetectable viral load. During her hospitalization, an acute renal failure with levels of creatininemia at 200 *μ*mol/L (2.3 mg/dL) led investigation of the urinary tract. In particular, an abdominal computed tomography (CT) demonstrated a 5 mm renal calculus with ureteral dilatation and renal hypotrophy involving a chronic obstruction. The radiodensity was low (<500 Hounsfield units) suggesting the presence of a uric acid stone (Figure 1). Owing to COVID-19 infection and comorbidities, a nonsurgical approach was particularly desirable as first-line treatment and medical dissolution therapy (consisting of urine alkalinization with potassium citrate) was started. Six weeks later, a repeat CT scan of the kidneys confirmed the persistence of the stone without improvement of renal function, and an extraction by ureteroscopy was required. Fragments of the stone were sent to the laboratory for constitutional analysis by Fourier transform infrared (FTIR) spectroscopy.

**Figure 2:**
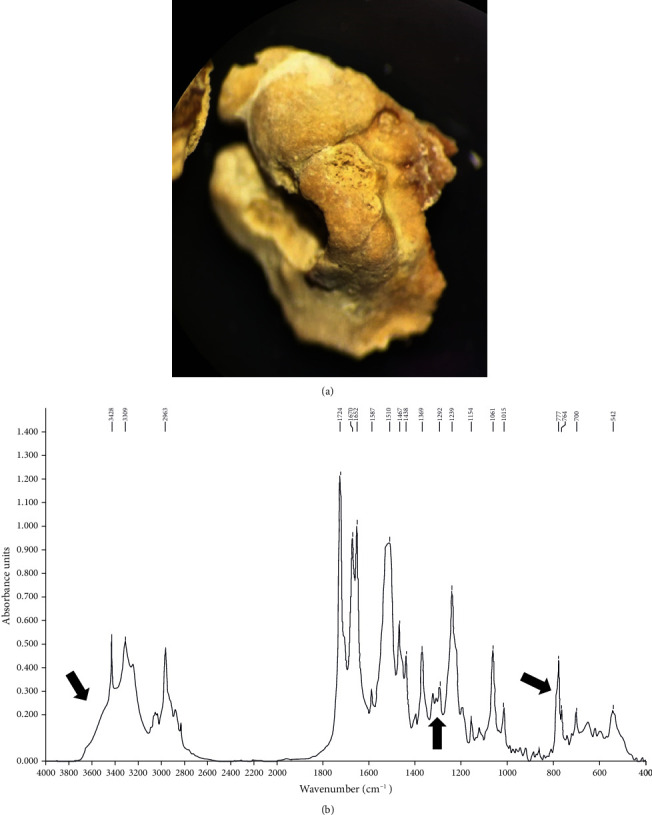
(a) Morphological analysis showed unusual fragments, yellowish in color, finely rough, and friable. Cross-section was relatively well organized, with porous structure and radial crystallization locations. (b) Infrared spectrum of atazanavir, C1, and proteins observed after crushing the stone and FTIR spectroscopy analysis (Bruker Optics, Germany). Arrows indicate places where C1 is identifiable in the main spectrum of atazanavir.

FTIR analysis revealed that the calculus was predominantly constituted of atazanavir (>70%), with a low proportion of monohydrated calcium oxalate (C1) and proteins (<30%) (Figures 2(a) and 2(b)). The spectrum of pure atazanavir is available in the supplementary figure (supplementary figure 1). These observations imply that the renal stone appeared to be entirely due to a drug-induced stone of atazanavir developed concomitantly with episodes of intermittent hyperoxaluria. Unfortunately, HPLC/LCMS analysis of the stone material in a multi-HIV drug test to definitively prove the stone's identity could not be performed. Our patient's medication history showed that ritonavir-boosted atazanavir (ATV/r) associated with tenofovir was carried on from 2007 to 2009. In 2009, tenofovir was switched to abacavir, and association ATV/r with abacavir was given until 2017. After 2017, the patient switched to another combination with a unique tritherapy galenic form. Apart from atazanavir, no other molecule has been identified in the patient's medication history likely to be the cause of the stone. The family investigation showed no personal or family history of urolithiasis.

## 3. Discussion

The analysis of the stone alone makes the diagnosis of drug-induced urolithiasis uncertain. The patient's medical history and the clinical context should be carefully considered to assess the degree of causality of the molecule. For example, other antiretroviral molecules (indinavir or other protease inhibitors) and antiviral molecules (acyclovir and foscarnet) but also antibacterial molecules (such as ampicillin, amoxicillin, ciprofloxacin, ceftriaxone, or vancomycin) are known to crystallize in urine under specific conditions and initiate drug stone formation. Moreover, few other mechanisms related to the intake of certain medications (vitamin D + calcium, alkalizing molecules, and carbonic anhydrase inhibitors) can induce kidney stones with a metabolic appearance, mainly constituted of oxalate/calcium phosphate and radiopaque. In this case, their prevalence is certainly underestimated [3].

As illustrated in the present report, the ureteral stone was discovered four years after the ATZ discontinuation administered during eight years. The CT scan suggested that the urinary tract obstruction was chronic and that the episode of acute renal failure was more functional. Although it is difficult to assess the degree of causality of the drug, a drug-induced urolithiasis should be considered when renal function declines even far from discontinuation of atazanavir.

In the last decade, a few case reports and small retrospective studies reported on renal stones related to atazanavir drug intake. HIV treatment changes rapidly, and a history of anti-HIV medication is essential to establish a causal link between taking medication and kidney stones. The causality is all the more difficult to establish notably when nephrolithiasis is revealed after several years of drug intake.

The reason why atazanavir treatment is associated with kidney stones is still unknown, but previous works described that urolithiasis recurrence, a ritonavir-boosted atazanavir (ATV/r) long exposure, or high levels of bilirubinemia are all related factors of atazanavir urolithiasis [4, 5]. Another study concluded that discontinuation of tenofovir could induce urolithiasis in ATV/r treatment by increasing concentration of atazanavir [6]. In a retrospective study conducted on 1134 patients (11 of whom having developed urolithiasis), the mean interval between the start of ATV/r therapy and the symptoms of urolithiasis was reported to be of 1.9 years [2]. Obviously, it is very difficult to establish when the patient stone formation begins, but in the present case, we can assume that the stone was formed at least 10 years earlier for two reasons. Firstly, the patient had a long duration of ATV/r treatment, and also, the discontinuation of tenofovir is suggested to be a trigger for crystallization of atazanavir in urine. Furthermore, identification of risk factors should alert to the possibility of drug-induced nephrolithiasis. Previous work reported that prolonged exposure to atazanavir is a risk factor for urinary crystallization. Such crystals are found in 8.9% of asymptomatic patients, all taking ritonavir-boosted atazanavir [7]. Moreover, crystal deposits appear to be a trigger for some renal deleterious changes, such as interstitial nephritis and urolithiasis [1]. Urinalysis including crystalluria during atazanavir long exposure may be performed because it is the first step towards the formation of the calculus. Increased daily diuresis and a close monitoring of urine are thus essential to prevent these adverse events. Lastly, withdrawal of ATV/r treatment should be considered in patients who have already experienced urolithiasis.

## Data Availability

Data will be made available on request at m-plawecki@chu-montpellier.fr.

## Supplementary Materials

Supplementary Materials Supplementary Figure 1: spectrum of pure atazanavir (Centre de recherches et d'information scientifique et technique appliquées aux lithiases, Laboratoire Cristal, Stage analyse morphologique et infrarouge des calculs, Module 2: infra-rouge, M. Daudon, Bioformation).
