# Supplementary figures and images for: Drug-Induced Urinary Stone of Atazanavir Incidentally Found in an Asymptomatic Patient: A Case Report
Source: Case Rep Urol. 2023 Mar 29;2023:4890711. doi: 10.1155/2023/4890711 (PMC10076111; doi:10.1155/2023/4890711)

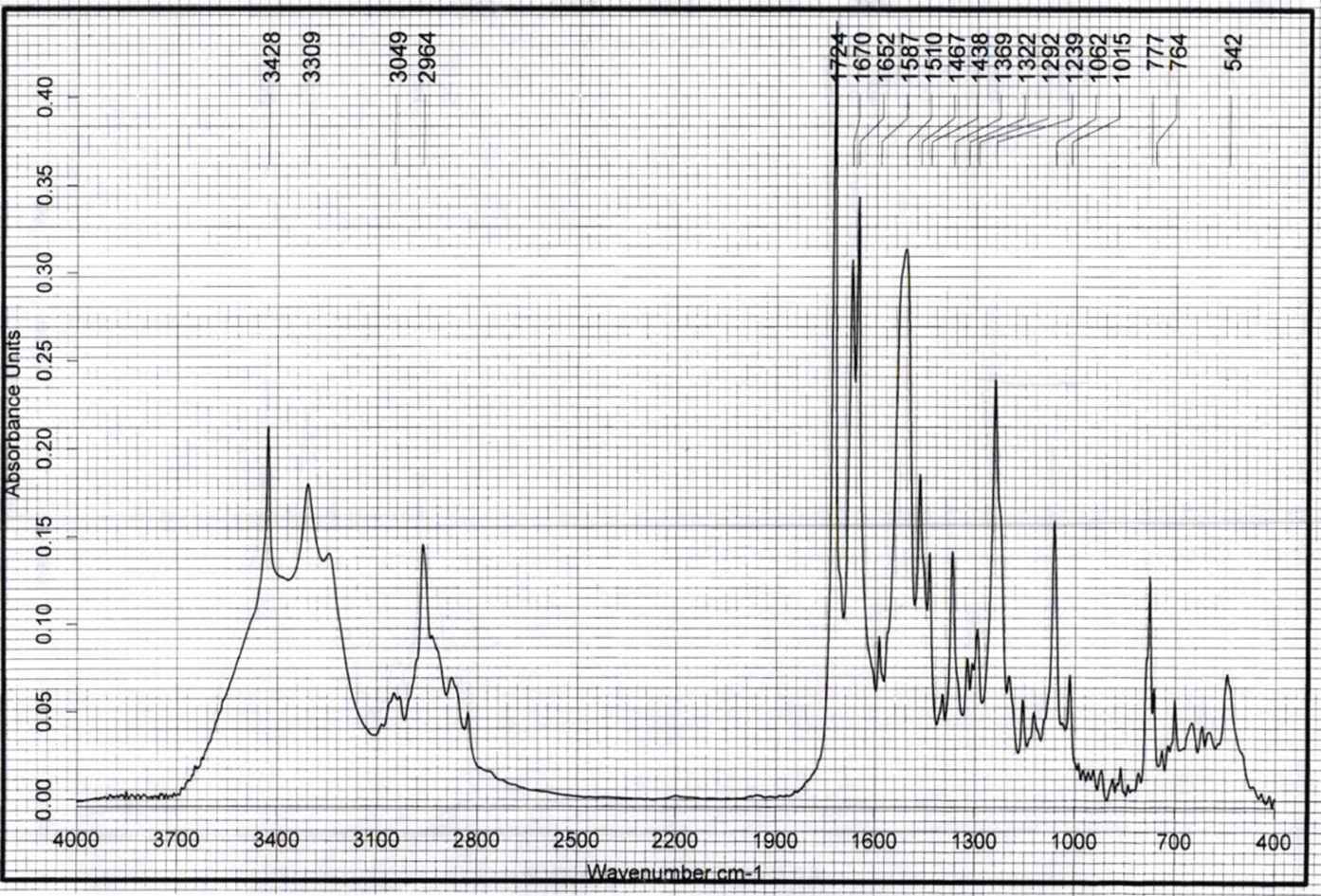

Supplement: Supplementary Materials — Supplementary Figure 1: spectrum of pure atazanavir (Centre de recherches et d'information scientifique et technique appliquées aux lithiases, Laboratoire Cristal, Stage analyse morphologique et infrarouge des calculs, Module 2: infra-rouge, M. Daudon, Bioformation). [file 4890711.f1.docx]
